# Comparative Transcriptome Analysis Reveals Coordinated Transcriptional Regulation of Central and Secondary Metabolism in the Trichomes of Cannabis Cultivars

**DOI:** 10.3390/ijms23158310

**Published:** 2022-07-27

**Authors:** Hock Chuan Yeo, Vaishnavi Amarr Reddy, Bong-Gyu Mun, Sing Hui Leong, Savitha Dhandapani, Sarojam Rajani, In-Cheol Jang

**Affiliations:** 1 Temasek Life Sciences Laboratory, 1 Research Link, National University of Singapore, Singapore 117604, Singapore; yeodynasty@yahoo.com.sg (H.C.Y.); vaishnavi@tll.org.sg (V.A.R.); mun830301@gmail.com (B.-G.M.); singhui@tll.org.sg (S.H.L.); savitha@tll.org.sg (S.D.); 2 Department of Biological Sciences, National University of Singapore, Singapore 117543, Singapore

**Keywords:** Cannabis, transcriptomics, cannabinoids, terpenes, MEP pathway, MVA pathway, trichomes

## Abstract

Cannabis is one of the few plant genera capable of producing cannabinoids, the effects of which are synergized by terpene interactions. As the biosynthesis of both metabolite classes requires the same intracellular feedstocks, this work describes the coordinated regulation of global metabolic pathways that allows for their joint copious production in vivo. To this end, a transcriptomics-based approach to characterize the glandular trichomes of five Cannabis cultivars was pursued. Besides revealing metabolic traits that enhanced and proportionated the supply of critical carbon precursors, in-depth analysis showed significantly increased gene expression of two particular enzymes to meet the huge nicotinamide adenine dinucleotide phosphate (NADPH) demand of secondary metabolite production. Furthermore, it led to a hypothesis that the methyl-d-erythritol 4-phosphate pathway might be utilized more than the mevalonic acid pathway in Cannabis trichomes. While both pathways were found to be activated in a modular and calibrated way that reflected their broad participation in physiological processes, the genes for hexanoate, cannabinoid, and terpene biosynthesis were, in contrast, up-regulated in an en bloc and multi-loci manner due to their specific roles in secondary metabolite production. In addition, three new terpene synthases were characterized based on both in silico and experimental assays. Altogether, the study enhances the current understanding of secondary metabolite production in Cannabis cultivars, which may assist in their characterization and development.

## 1. Introduction

Cannabis is a member of the plant family Cannabaceae. It encompasses three species, *Cannabis sativa*, *Cannabis indica*, and *Cannabis ruderalis*, which are of high medicinal and commercial value due to their production of therapeutic and psychoactive secondary metabolites. Although Cannabis was mainly consumed as an illegal drug in early years, due to its high (−)-*trans*-Δ^9^-tetrahydrocannabinol (THC) content, it is now recognized as a source of other non-psychoactive, therapeutic cannabinoids, such as cannabidiol (CBD) [1]. For example, Cannabis-based drugs have been shown to be effective for treating several disorders, such as Parkinson’s disease, epilepsy, schizophrenia, inflammatory bowel disease, oxidative stress, inflammation, and brain injury. A recent review listed various Cannabis-based or -inspired medicines and their pharmaceutical status [2].

Over the years, several natural Cannabis varieties and developed cultivars (pure or hybrid) have been used [3]. The legalization of Cannabis in many countries has led to increased demand, which provides further impetus for developing crops with desired cannabinoid profiles. Cannabinoid biosynthesis mainly occurs in the glandular trichome of female Cannabis inflorescences. Subcellularly, the pathway starts in the cytosol, where the precursor, hexanoic acid, is derived from the oxidative cleavage of fatty acids, such as palmitic acid, by an acylactivating enzyme (AAE) to form hexanoyl coenzyme A. The latter is then biochemically combined with three malonyl coenzyme A molecules by olivetol synthase and olivetolic acid cyclase (OAC) to form olivetolic acid (OA) [4]. Following this, the acid is prenylated to form cannabigerolic acid (CBGA) as the starting cannabinoid, using geranyl diphosphate (GPP) made via the chloroplastic MEP pathway [5]. Subsequent oxidative cyclization of CBGA in the resin cavity results in the production of various other cannabinoids [6]. Notably, the cannabinoids, tetrahydrocannabinolic acid (THCA) and cannabidiolic acid (CBDA), can also be decarboxylated by heating to produce THC and CBD, respectively [7]. In all, close to 120 cannabinoids have been identified [8,9], in 10 categories: (−)-Δ^8^-*trans*-tetrahydrocannabinols (Δ^8^-THC), (−)-Δ^9^-*trans*-tetrahydrocannabinols (Δ^9^-THC), CBD, cannabitriols (CBT), cannabigerols (CBG), cannabinols (CBN), cannabicyclols (CBL), cannabinodiols (CBND), cannabichromenes (CBC), and cannabielsoins (CBE) [10]. Although several cannabinoid biosynthetic enzymes have been identified [11,12,13], the genetics underlying their diversity in the plant remains poorly understood [14].

Similarly, terpenes are also biosynthesized and stored in large amounts in the glandular trichome of female Cannabis inflorescences. Some common terpenes produced by Cannabis include the monoterpenes, (R)-linalool, α-pinene, and limonene, as well as the sesquiterpenes, bisabolol, (E)-β-farnesene, β-caryophyllene, and α-humulene [15]. Some are thought to interact with cannabinoids and regulate or change their physiological effects [16]. As a result, such ‘entourage effect’ also modulates the medicinal uses of Cannabis plants [17]. For example, β-caryophyllene can be used as a dietary supplement due to its ability to enhance binding to the CB2 cannabinoid receptor, thus reducing gastrointestinal inflammation [18]. The metabolic precursors of terpenes, isopentenyl diphosphate (IPP) and its isomer, dimethylallyl diphosphate (DMAPP), are both produced by either the plastid-localized MEP pathway or the cytosolic MVA pathway. The pathways utilize glyceraldehyde-3-phosphate (G3P) and pyruvate as carbon feedstocks that are generated via plastid-localized glycolytic steps [19,20]. Following this, the condensation of single molecules of five-carbon IPP and DMAPP leads to the formation of a 10-carbon GPP molecule (which is also used for cannabinoid production). A similar process between GPP and IPP then results in a 15-carbon farnesyl diphosphate (FPP) moiety, which further condenses with IPP to form a 20-carbon geranylgeranyl diphosphate (GGPP) molecule. GPP, FPP, and GGPP are then acted upon by various terpene synthases (TPSs) to form monoterpenes, sesquiterpenes, and diterpenes, respectively. In this regard, monoterpenes and diterpenes are synthesized via the MEP pathway, whereas sesquiterpenes are made via the MVA pathway. Depending on the isoforms, GPP synthase (GPPS) and GGPP synthase (GGPPS) can be found in the chloroplasts, mitochondria, and endoplasmic reticulum [21,22], while FPP synthases (FPPS) are mostly cytosolic, although some are found in the peroxisome or endoplasmic reticulum [20]. As the MEP and MVA pathways are also used to provide precursors for primary metabolism and hormone biosynthesis [23], depending on developmental, environmental, and genetic factors, it is imperative to characterize them alongside cannabinoid and terpene biosynthetic pathways in Cannabis plants for the selection and development of cultivars.

Although it is generally accepted that genetic regulatory adaptations exist in the trichomes to facilitate secondary metabolite production across diverse plant species, most studies in Cannabis to date have focused on secondary metabolism. However, we hypothesized a more holistic and coordinated adjustment of the metabolism, including the primary pathways, that are also reflected at the transcriptome level. To test the hypothesis, the transcriptome of female inflorescence trichomes from five different Cannabis cultivars were profiled and then compared to a non-trichome tissue, namely, stem tissue (stripped of any existing trichomes), to identify differentially expressed genes (DEGs) or those genes specific to trichomes that are broadly conserved among the different cultivars. The expression of biosynthetic enzymes in the central and secondary metabolic pathways in the trichomes was then examined and interpreted in terms of functional coordination to enhance cannabinoid and terpene production. Furthermore, their levels were compared among the cultivars to provide the molecular basis for characterizing their yield variations. Additionally, three TPSs were functionally characterized using in vitro and in vivo assays. Altogether, these findings offer opportunities for the selection and improvement of yield profiles in Cannabis cultivars.

## 2. Results

### 2.1. RNA-seq-Based Approach for Deciphering Conserved Expressions of Metabolic Enzymes

**Figure 1:**
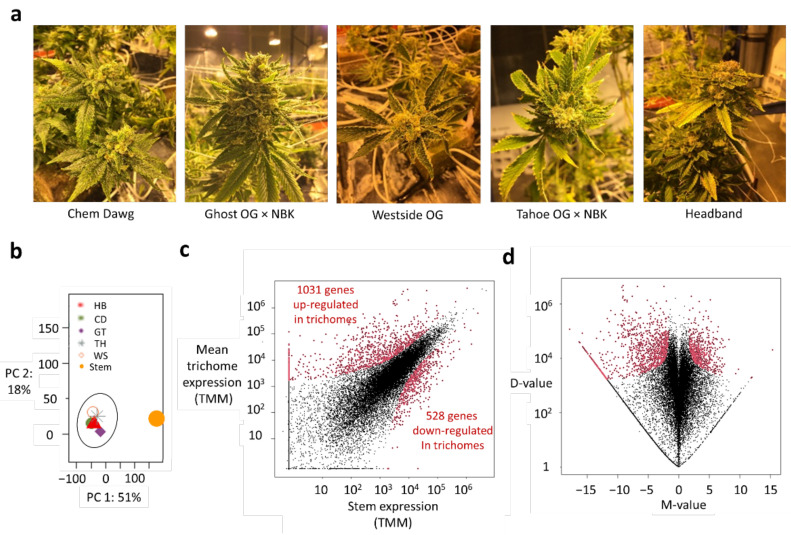
Quality assessment and differential analysis of gene expression data. (**a**) Picture of Cannabis cultivars selected for investigation. (**b**) Score plot of trichome and stem samples according to the first and second components (PC 1 and PC 2) obtained from a principal component analysis. (**c**) Plot of average gene expression values for the trichomes of five cultivars versus the corresponding expression in the stem tissue of HB. (**d**) M-D plot of global gene expression data. (M-value: log_2_ FC value; D-value: absolute-difference-in-expression value). Red dots in (**c**,**d**) denote genes that are differentially expressed. HB: Headband; CD: Chem Dawg; GT: Ghost OG × NBK; TH: Tahoe OG × NBK; WS: Westside OG.

To elucidate the transcriptional regulation of both primary and secondary metabolic pathways in the trichomes of the Cannabis plant, an RNA-sequencing (RNA-seq) approach was pursued. Five Cannabis cultivars, Chemdawg (CD), Ghost OG × NBK (nothing but Kush) (GT), Westside OG (WS), Tahoe OG × NBK (TH), and Headband (HB), were selected for investigation, as they are commonly planted (Figure 1a). CD is a sativa cultivar, while GT, WS, TH, and HB, are indica-dominant cultivars. All of them are known to produce high levels of THC, along with lesser amounts of CBD and other minor cannabinoids. The transcriptome of the trichomes was compared with that of a representative stem of the HB cultivar, whose trichomes were removed entirely. Furthermore, differences in the biosynthetic pathways among the trichomes of the cultivars were picked out to provide the molecular basis for profiling their yield variations.

RNA-seq yielded between 248 to 277 million raw reads in both RNA strand directions per sample (Table 1), with 3–8% of the reads subsequently removed by pre-processing and quality-control measures. The resulting reads were quasi-mapped to the latest reference transcriptome of *Cannabis sativa* (GenBank assembly accession: GCA_900626175.2) for the generation of transcript read counts and further corrected for sequencing biases. In the process, between 200 to 235 million reads were used to tabulate the transcripts in each sample, representing 86–88% of the initial raw reads. The read counts of the transcripts were then added up for each gene. As a result, a total of 22,578 genes had reads mapped to their sequences in at least one sample. After further data normalization to enable sample and gene comparisons, the data quality was assessed using principal component analysis, which revealed a tight clustering of trichome samples away from the stem sample, indicating the absence of any trichome outliers (Figure 1b). For each cultivar, the global gene expression values of the trichomes were then plotted against those of stem tissue using a scatter plot, revealing their broadly similar distribution vis-à-vis the stem tissue: most genes lay near the diagonal through the origin and were thus not differentially expressed, as should be the case after normalization (Figure S1). Their alikeness was also apparent from the plot of absolute-difference-in-expression values (D-values) versus log_2_ FC values (M-values), thereby confirming the broad consistency in data quality among the cultivars. A subsequent hierarchical clustering of the M-values of DEGs further revealed regions of similar expression patterns between the GT and TH cultivars, and between WS and TH, with the former being possibly attributed to their common NBK ancestry (Figure S2a). On the other hand, CD and HB showed distinctive profiles. Overall, the five cultivars had about half of their DEGs in common, with 10% (or fewer) unique to each cultivar (Figure S2b, Table S3). Taken together, the observations suggested a moderate degree of diversity among the cultivars. Expectedly, a sizable percentage of the common DEGs encode for metabolic enzymes (40%), molecular transporters (16%), and transcription factors (5%), among other things (Table S4). To identify the regulation of metabolic enzymes more robustly, gene expressions of the trichomes for various cultivars were then jointly compared with the reference stem sample. As a result, 1031 and 528 genes were broadly identified as being up- and down-regulated in the trichomes, respectively (Figure 1c). Their D-values were generally greater than 1700, while their M-values were smaller or greater than −1.6 and 1.6, respectively (Figure 1d; Table S5).

### 2.2. Transcriptional Regulation of the Central Metabolism in Trichomes for Enhancing Carbon Feedstock Production

**Figure 2:**
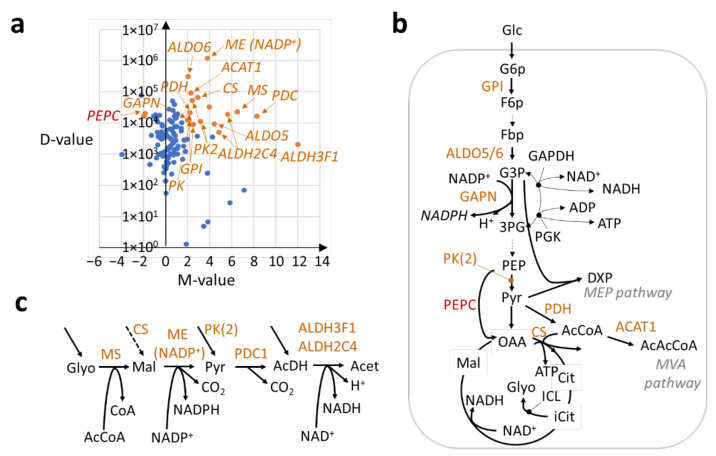
Transcriptional regulation of central metabolism in the trichomes of Cannabis cultivars with respect to stem tissue. (**a**) M-D plot of mean expression values (TMM) of genes encoding enzymes involved in the central metabolism (the glycolytic pathway, pyruvate metabolism, the pentose phosphate pathway, and the tricarboxylic acid cycle). Filled orange circles denote differentially up-regulated genes of interest, while filled blue circles represent all other genes in the central metabolism. It should be noted that *PEPC* is significantly down-regulated. (**b**) Pathway diagram of the central metabolism with corresponding enzyme names marked in red/orange. Note that ICL had very low expression. (**c**) Similar diagram for NAD(P)H replenishment pathway. Refer to Table S8 for corresponding gene IDs of enzymes. **Enzyme abbreviations in numeric and alphabetical order**: ACAT1 (acetyl-CoA acetyltransferase cytosolic 1); ALDH2C4 (aldehyde dehydrogenase family 2 member C4, NADH producing [-like]); ALDH3F1 (aldehyde dehydrogenase family 3 member F1, NADH producing); ALDO6 (fructose-bisphosphate aldolase 6, cytosolic); ALDO5 (fructose-bisphosphate aldolase 5, cytosolic); CS (ATP-citrate synthase); GAPDH (glyceraldehyde-3-phosphate dehydrogenase); GAPN (NADP-dependent glyceraldehyde-3-phosphate dehydrogenase); GPI-like (glucose-6-phosphate isomerase, chloroplastic-like); ICL (isocitrate lyase); ME (NADP^+^) (NADP-dependent malic enzyme); MS (malate synthase, glyoxysomal); PDC1-like (pyruvate decarboxylase 1-like); PDH (pyruvate dehydrogenase, chloroplastic); PEP (phosphoenolpyruvate carboxylase, housekeeping isozyme); PGK (phosphoglycerate kinase); PK (pyruvate kinase, cytosolic); PK2 (plastidial pyruvate kinase 2). **Metabolite abbreviations in numeric and alphabetical order**: 3PG (3-phosphoglyceric acid); AcCoA (Acetyl co-enzyme A); AcAcCoA (Acetoacetyl CoA); AcDH (Acetaldehyde); Acet (acetate); ADP (adenosine diphosphate); ATP (adenosine triphosphate); citrate (Cit); CoA (coenzyme A); DXP (1-deoxy-d-xylulose 5-phosphate); F6p (fructose-6-phosphate); Fbp (Fructose bisphosphatase); G3P (glyceraldehyde-3-phosphate); G6p (Glucose-6-phosphate); Glc (glucose); Glyo (glyolate); iCit (isocitrate); Mal (S-malate); NAD^+^ (Nicotinamide adenine dinucleotide); NADH (reduced NAD); NADP^+^ (NAD phosphate); NADPH (reduced NAD phosphate); OAA (oxaloacetate); PEP (phosphoenolpyruvate); Pyr (pyruvate).

Differential gene expression statistics were then mapped to the metabolic pathways to examine the likely effects of their transcriptional regulation on cannabinoid and terpenoid production. Most gene expression encoding enzymes in the central metabolism were up-regulated, as exemplified by the disproportionately larger number of positive M-values compared to negative ones (Figure 2a). Upon further analysis, the glycolytic pathway in the trichomes was found to be re-routed in a consequential manner compared to the stem, due to a significant increase in the expression of specific genes (Figure 2b). For example, the genome-wide expression of genes encoding the glucose-6-phosphate isomerase (GPI) enzyme increased by 73% overall (D = 2.09 × 10^4^, Table S6), thus allowing more glucose to be channeled into the pathway. In addition, a 154% net increase in the gene expression for fructose-bisphosphate aldolase (ALDO, D = 3.71 × 10^5^, Table S6) implied a markedly enhanced availability of G3P metabolites to the MEP pathway for cannabinoid and terpene production. G3P can also be used to produce more NADPH via higher expression of the gene encoding the glyceraldehyde-3-phosphate dehydrogenase [NADP+] (GAPN) enzyme (log_2_FC = 2, D = 1.23 × 10^4^) to meet the increased demand of the cofactors during secondary metabolite production (Figure 2b). The enhanced expression of *GAPN* in the trichomes has been verified by qPCR (Figure S3). On the other hand, the alternative production of NADH and ATP using G3P, through the action of glyceraldehyde-3-phosphate dehydrogenase (GAPDH) and phosphoglycerate kinase (PGK), was found to be suppressed by the down-regulation of the PGK-encoding gene in the trichomes (Figure 2b). This suggests a shift from NADH and ATP production to that of NADPH in the trichomes to support secondary metabolism.

Besides G3P and NADPH, pyruvate formation was also found to be promoted in the trichomes. There was a significantly higher expression of genes for pyruvate kinase (PK) enzymes (Figure 2b) that produce pyruvate, with an overall 78% increase in expression (D = 7.69 × 10^4^, Table S6). In further coordination, the phosphoenolpyruvate carboxylase (PEPC) enzyme was also down-regulated (log_2_FC = −1.9, D = 1.99 × 10^4^) to redirect phosphoenolpyruvate to pyruvate conversion, instead of oxaloacetate production. Pyruvate is also used as a substrate for forming acetyl coenzyme A (AcCoA) via pyruvate dehydrogenase (PDH) reactions [24]. Consistently, gene expression encoding a component of PDH (dihydrolipoyllysine-residue acetyltransferase component 5) was found to be up-regulated in the trichomes, with a corresponding log_2_ FC value of 2.2, representing an increase of 2.71 × 10^4^ in the ‘Trimmed Means of M-values’ (TMM) expression (Figure 2b). In turn, the AcCoA was in high stoichiometric demand by the MVA pathway and for the biosynthesis of malonyl coenzyme A and hexanoyl coenzyme A, all of which are needed to produce cannabinoids and terpenes. Further, AcCoA was found to be essential for replenishing the tricarboxylic acid (TCA) cycle in the trichomes, as evidenced by the up-regulation of the ATP-citrate synthase (CS) enzyme in comparison to stem tissue (log_2_FC = 2.9, D = 6.64 × 10^4^, Figure 2b). Altogether, the importance of enhancing and tuning the balance between four intracellular feedstocks—NADPH, G3P, pyruvate, and AcCoA—in the central metabolism for promoting the production of secondary metabolites in the trichomes was uncovered.

### 2.3. An Uncovered Pathway for Meeting NAD(P)H Demand in Trichomes

Besides GAPN, the current analysis further implicated the transcriptional amplification of four consecutive reactions catalyzed by malate synthase (MS), malic enzyme [NADP+ dependent] (ME [NADP+]), pyruvate decarboxylase 1 (PDC1), and aldehyde dehydrogenases (ALDH) to further meet the demand of NAD(P)H cofactors for secondary metabolite production in the trichomes (Figure 2c). The pathway is as follows: the MS enzyme first transfers the acetyl moiety of AcCoA to glyoxylate for the formation of malate. In turn, malate, which is also well replenished by the TCA cycle due to enhanced *CS* expression (Figure S3), is oxidized by ME (NADP+) in the second reaction to form pyruvate, as well as the first NADPH molecule produced by the pathway. In the third reaction, pyruvate, which is also well provided by glycolysis, is then oxidized by PDC1 to form acetaldehyde. The resulting aldehyde molecule can then be oxidized by ALDHs (ALDH2C4 (ALDH family 2 member C4) and ALDH3F1 (ALDH family 3 member F1)) to generate the second NADH molecule as well as acetate (Figure 2c). Besides being major cellular sources of reducing equivalents through NAD(P)H production [25], ALDHs are also required for generating hexanoic acid, a key precursor for cannabinoid production [4]. Importantly, the pathway prioritized NADPH production over NADH, which was similarly observed with the relative regulation of GAPN, GAPDH, and PGK, as described previously. For example, while the fold-change value of the pathway enzymes was generally large (log_2_FC = 3.8 to 8.3) compared to stem tissue, the ME (NADP+) enzyme stood out in having an increased expression (D) that was at least 50 times those of the three individual pathway enzymes (1.2 × 10^6^ versus 1.7 × 10^4^–2.3 × 10^4^) and close to a hundred times that of GAPN (1.23 × 10^4^) (Figure S3). This observation thus implicates ME (NADP+) as another major contributor to NADPH production in trichome cells, besides GAPN.

### 2.4. Modular and Calibrated Regulation of MEP and MVA Pathways

**Figure 3:**
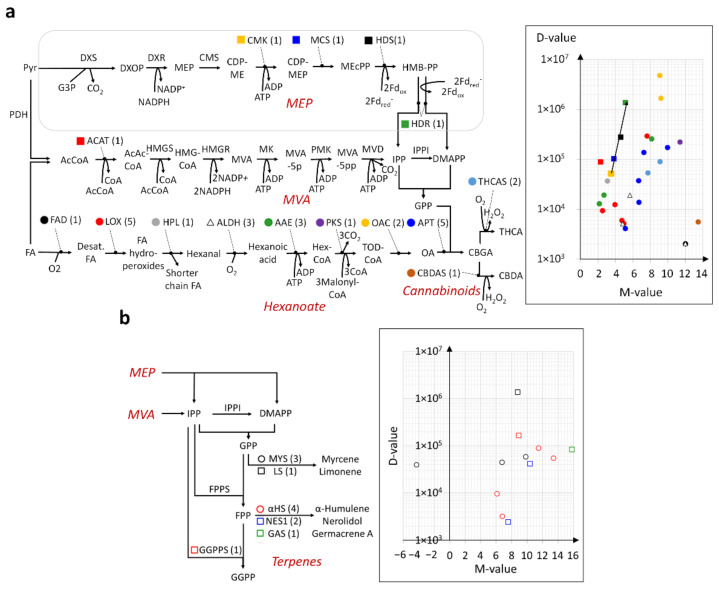
Distinct mode of transcriptional regulation for secondary metabolite biosynthetic pathways. (**a**) M-D plot of DEGs (trichomes versus stem tissue) in the MEP, MVA, hexanoate, and cannabinoid pathways, with corresponding enzyme names being similarly color-coded in the pathway schematic on the left. The numbers inside brackets indicate the numbers of DEGs. Note that the black arrow in the M-D plot illustrates the increasing gene regulation at the end of the MEP pathway. (**b**) Similar M-D plot and corresponding enzyme names in the context of the terpene biosynthetic pathways. Refer to Table S8 for corresponding gene IDs of enzymes. **Enzyme abbreviations for MEP pathway** (left to right): DXS (DOXP synthase); DXR (DXP reductoisomerase); CMS (2-C-methyl-d-erythritol 4-phosphate cytidylyltransferase); CMK (4-diphosphocytidyl-2-C-methyl-d-erythritol kinase); MCS (2-C-methyl-d-erythritol 2,4-cyclodiphosphate synthase); HDS (HMB-PP synthase); HDR (HMB-PP reductase), **MVA pathway** (left to right): ACAT (acetyl-coenzyme A acetyltransferase); HMGS (HMG-CoA synthase); HMGR (HMG-CoA reductase); MK (mevalonate-5-kinase); PMK (phosphomevalonate kinase); MVD (mevalonate-5-pyrophosphate decarboxylase); IPPI (isopentenyl diphosphate isomerase); GPPS (GPP synthase), **Hexanoate pathway** (left to right): FAD (fatty acid desaturase); LOX (lipoxygenase); HPL (hydroperoxide lyase); ALDH (aldehyde dehydrogenase); AAE (acyl activating enzyme); PKS (polyketide synthase); OAC (olivetolic acid cyclase), **Cannabinoid pathway**: APT (aromatic prenyl transferase); THCAS (THCA synthase); CBDAS (CBDA synthase), **Terpene pathway** (top to bottom): MYS (myrcene synthase); LS (limonene synthase); FPPS (Farnesyl diphosphate synthase); αHS (α-humulene synthase); NES1 (nerolidol synthase); GAS (Germacrene A synthase); GGPPS (geranylgeranyl diphosphate synthase). **Metabolite abbreviations in numeric and alphabetical order:** AcCoA (Acetyl co-enzyme A); AcAcCoA (Acetoacetyl CoA); ADP (adenosine diphosphate); ATP (adenosine triphosphate); CBDA (cannabidiolic acid); CBGA (cannabigerolic acid); CDP-ME (4-diphosphocytidyl-2-C-methylerythritol); CDP-MEP (4-diphosphocytidyl-2-C-methyl-d-erythritol 2-phosphate); CoA (coenzyme A); DMAP (Dimethylallyl pyrophosphate); DXOP (deoxyxylulose 5-phosphate); FA (fatty acids); F_dox_ (oxidized ferredoxin); Fd_red-_ (reduced ferredoxin); FPP (Farnesyl diphosphate); G3P (glyceraldehyde-3-phosphate); GGPP (geranylgeranyl diphosphate); GPP (geranyl diphosphate); Hex-CoA (hexanoyl-CoA); HMB-PP ([E]-4-Hydroxy-3-methyl-but-2-enyl pyrophosphate); HMG-CoA (3-hydroxy-3-methylglutaryl-CoA); IPP (isopentenyl diphosphate); MEcPP (2-C-methyl-d-erythritol 2,4-cyclodiphosphate); MEP (2-C-methyl-d-erythritol 4-phosphate); MVA (mevalonate); MVA-5p (mevalonate-5-phosphate); MVA-5p (mevalonate-5-diphosphate); NADP^+^ (Nicotinamide adenine dinucleotide phosphate); NADPH (reduced nicotinamide adenine dinucleotide phosphate); OA (olivetolic acid); Pyr (pyruvate); TOD-CoA (trans octadecenoyl CoA); THCA (tetrahydrocannabinolic acid).

There are also notable features regarding the regulation of MEP and MVA pathways. Firstly, the regulation of the MEP pathway appeared to be modular and calibrated with the up-regulation of four successive end reactions in an increasing manner, i.e., both log_2_FC and D-values increased in the order of 4-diphosphocytidyl-2-C-methyl-d-erythritol kinase (CMK), 2-C-methyl-d-erythritol 2,4-cyclodiphosphate synthase (MCS), 4-hydroxy-3-methylbut-2-en-1-yl diphosphate synthase [ferredoxin] (HDS), and 4-hydroxy-3-methylbut-2-enyl diphosphate reductase (HDR) (Figure 3a and Figure S4). Among them, HDS and HDR are known to be rate-limiting [26], and, as such, their regulation is expected to enhance the sensitivity of tuning the pathway. Figure S3 shows the up-regulated expression of *MCS*, *HDS* and *HDR* in the trichomes of all varieties compared to the stem. In this regard, the current finding provides fresh evidence that they serve as control points in the pathway [27]. On the other hand, in the MVA pathway, only the initial fast reaction catalyzed by acetyl-coenzyme A acetyltransferase 1 (ACAT1) was found to be significantly increased in terms of gene expression (Figure 2b, Figure 3a, and Figure S3), thus suggesting a limited increase in the utilization of the pathway. Overall, the modular and calibrated mode of regulation for both pathways might be due to their broader role in integrating environmental and developmental cues, thus necessitating the careful and effective adjustment of pathway activity by specialized modules [27].

### 2.5. En Bloc and Multi-Loci Up-Regulation of the Hexanoate Pathway and Cannabinoid Biosynthesis

The regulation of the hexanoate pathway was distinct from the MEP and MVA pathways in two ways: (i) the en bloc transcriptional up-regulation of all seven steps in the pathway, which is frequently achieved via (ii) numerous gene loci (Figure 3a). Such multi-loci regulated enzymes included linoleate lipoxygenase (LOX), ALDH, AAE, and OAC, with their log_2_ FC values of gene expression ranging from 2.5 to 7.6 (5 loci), 4.8 to 12 (3 loci), 2.1 to 8.1 (3 loci), and 9.1 to 9.2 (2 loci), respectively. This mode of regulation was also found with enzymes directly involved in cannabinoid biosynthesis (Figure 3a). For example, the aromatic prenyltransferases (APT) and THCA synthase (THCAS) enzyme genes showed respective log_2_ FC values ranging between 5.1 and 9.9 (5 loci), and 7.7 and 91 (2 loci). The en bloc and multigene loci mode of regulation shared by both pathways can be ascribed to their more straightforward role in cannabinoid production in the trichomes compared to the MEP and MVA pathways.

### 2.6. Metabolic Profiles of Cultivars

**Figure 4:**
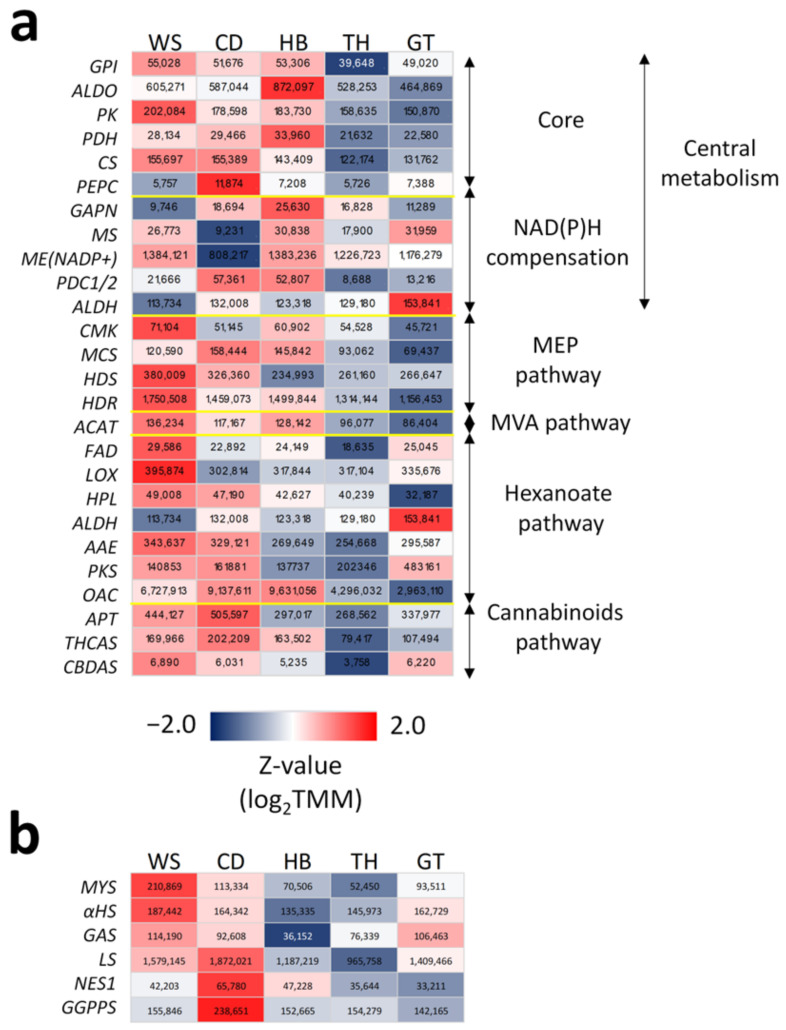
Biosynthetic profiles of Cannabis cultivars. (**a**) Cultivar-normalized expression heatmap of significantly regulated genes encoding metabolic enzymes involved in cannabinoid and terpene biosynthesis. The heatmap is based on the total expression values of genes that encode enzymes with the same products. The total gene expression values (TMM as units) are also given in each cell. Note that *ALDH* is associated with both NAD(P)H compensation and the hexanoate pathway. (**b**) Similar heatmap for key TPSs, including those shortlisted for functional validation. For the full names for all enzymes and metabolites, refer to Figure 1 and Figure 2. Refer to Table S8 for the gene IDs involved.

Furthermore, the gene expressions of the metabolic enzymes were compared among the cultivars to elucidate their relative production capacities. Most genes in the core central metabolism, as well as MEP and MVA pathways, were highly expressed in the WS cultivar, unlike others, suggesting that it has the highest NADPH and carbon feedstock productivities (Figure 4a). Consistently, the WS cultivar also had one of the lowest gene expressions for the PEPC enzyme, thus enabling it to redirect phosphoenolpyruvate to pyruvate feedstock production. Additionally, in the WS cultivar, the low gene expression for PDC and ALDH enzymes that drive NADH production (Figure 2c) may be compensated by high CS expression replenishing the TCA cycle to make the cofactors. Similarly, the high expression of CS and *ME (NADP+)* in WS may make up for its relatively low *GAPN* expression in producing NADPH. On the other hand, the HB cultivar seemed to excel in NADPH production via the up-regulation of both *GAPN* and *ME* (*NADP+*) genes, but was partly offset by its lower *CS* expression level compared to the WS cultivar. The WS cultivar also had broadly higher gene expressions in the hexanoate pathway (Figure 4a), suggesting its larger upstream capacity for cannabinoid production. In this regard, its lower *ALDH* level could already be sufficient for generating the required hexanoic acid for synthesizing cannabinoids (Figure 3a). Both the WS and CD cultivars also had some of the highest transcript abundances for APT, THCAS, and CBDAS enzymes but were still somewhat matched by the HB cultivar for THCAS and by the GT cultivar for CBDAS. Notably, the TH cultivar showed relatively low gene expression levels across all metabolic pathways (Figure 4a).

Expectedly, the gene expression for five major TPSs, namely, myrcene synthase (MYS), limonene synthase (LS), α-humulene synthase (αHS), nerolidol synthase (NES), and germacrene A synthase (GAS), were significantly up-regulated in the trichomes compared to the stem (Figure 3b and Figure S4). The CD cultivar had the highest total transcript levels for *LS* (TMM = 1.87 × 10^6^) and *NES* (6.58 × 10^4^), which were about twice those of the TH cultivar with the lowest expression (TMM = 9.66 × 10^5^ and 3.32 × 10^4^, respectively) (Figure 4b). On the other hand, the WS cultivar had the highest gene expression for *MYS*, *αHS*, and *GAS*.

As no chemical profiling data were available for rationalizing the metabolic outputs based on gene expression, publicly available information was used for the few comparisons that were possible (Table S7). Although THC levels were indeed higher in both WS and CD cultivars compared to others, as would be expected from their *THCAS* expression (Figure 4a), the levels of cannabinoids were, however, not in line with the relative gene expression of *THCAS* in WS and CD. CBDA and β-myrcene were also expectedly higher in WS compared to CD, based on respective *CBDAS* and *MYS* expressions (Figure 4b). On the other hand, the relative levels of limonene in WS and CD cultivars were inconsistent with the relative expressions of *LS* in the two cultivars.

### 2.7. Functional Characterization of New TPSs

Besides previously reported TPSs from Cannabis [28,29,30,31], three new TPSs were identified from the RNA-seq data containing full-length open reading frames (ORFs). They were designated as *CsTPS3GT*, *CsTPS4WS*, and *CsTPS5TH*, to reflect their respective origins in the GT, WS, and TH cultivars. *CsTPS4WS* and *CsTPS5TH* had very high expressions in the trichomes of all cultivars, whereas *CsTPS3GT* showed lower expression in the trichomes of the HB, CD, and WS cultivars compared to the stem (Figure S5). Based on amino acid sequence similarity, CsTPS3GT was 96% similar to CsTPS31 (accession number: QLC36839.1), which was previously characterized as producing an unknown sesquiterpene with an RI of 1916 and a base peak of 93 [29]. Furthermore, CsTPS4WS was identical to CsTPS29 (accession number: QLC36833.1), which was previously identified as a linalool synthase [29], while CsTPS5TH was 98.7% similar to CsTPS35 (accession number: QLC36840.1), a linalool/nerolidol synthase [29].

**Figure 5:**
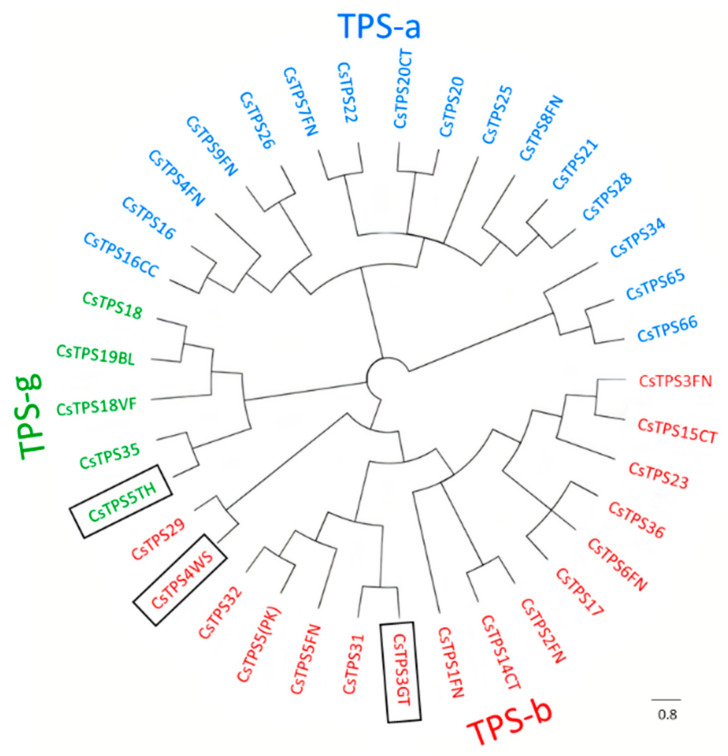
Phylogenetic analysis of CsTPSs. Neighbour-joining phylogenetic tree of CsTPS3GT, CsTPS4WS, and CsTPS5TH, with other CsTPSs. The scale bar indicates the number of amino acid substitutions per site.

As expected for TPSs, all three peptides contain the ‘DDXXD’ and ‘NSE/DTE’ motifs, which allow them to participate in divalent metal ion-assisted binding of substrates and cofactors [32]. Furthermore, both CsTPS3GT and CsTPS4WS contained a tandem arginine/tryptophan motif, ‘RR(X8)W’, at the N-terminal region (Figure S6), which could be used for monoterpene cyclization [33]. However, the plastid-targeting peptide, which is unique to mono-TPSs, was absent in CsTPS3GT (Figure S6). Phylogenetic analysis placed CsTPS3GT and CsTPS4WS in the TPS-b sub-family, whereas CsTPS5TH fell under the TPS-g sub-family (Figure 5).

**Figure 6:**
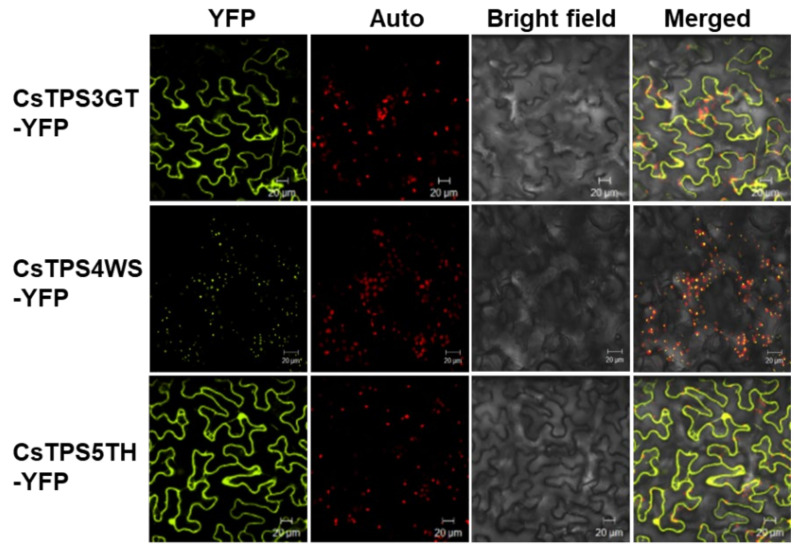
Subcellular localization of CsTPSs in *N. benthamiana* leaf cells. *A. tumefaciens* harbouring YFP-tagged CsTPS was infiltrated into *N. benthamiana* leaves. Then, the infiltrated leaves were visualized using a confocal microscope (2 dpi). Auto: chlorophyll autofluorescence; YFP: YFP channel image; Bright field: light microscope image; Merge: merged image between autofluorescence, YFP, and light channel. Scale bars: 20 μm.

The intracellular localization of each TPS was investigated in *N. benthamiana* leaf cells. Upon transient expression of YFP-tagged TPSs in the leaf cells, both CsTPS3GT and CsTPS5TH were found to localize to the cytosol (Figure 6), which is consistent with their lack of transit peptide sequences and thus suggests that they are sesqui-TPSs, rather than mono-TPSs (Figure 6). On the other hand, CsTPS4WS was localized to the chloroplasts, indicating it to be a mono-TPS.

### 2.8. In Vitro and In Vivo Identification of CsTPSs

**Figure 7:**
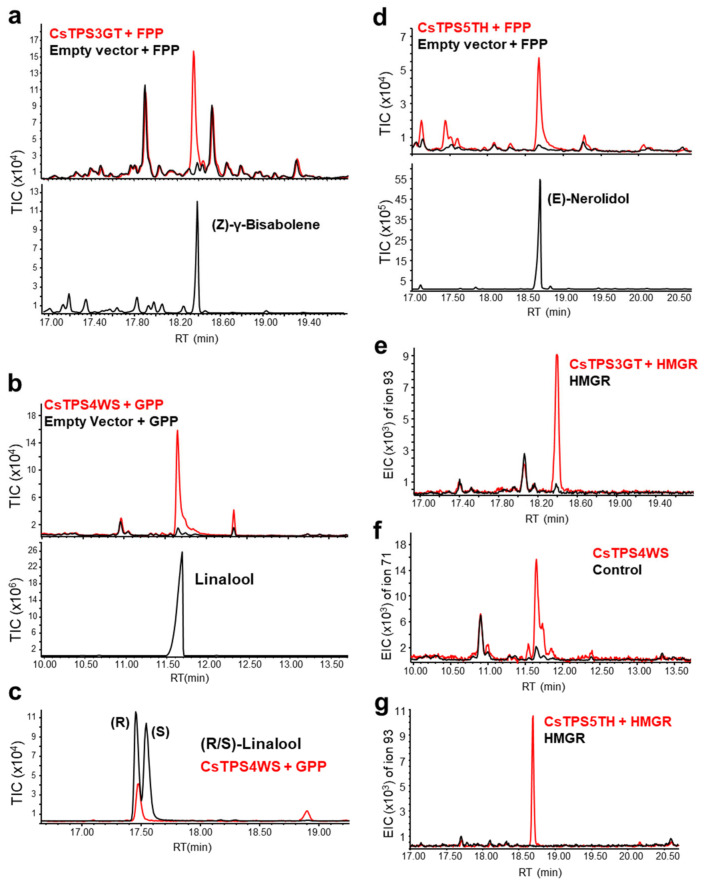
Functional characterization of CsTPSs. In vitro (**a**–**d**) and in vivo (**e**–**g**) characterization of CsTPS3GT, CsTPS4WS, and CsTPS5TH. The products were confirmed by comparison of retention times and mass spectra with those of standards (Figure S7). TIC: total ion chromatogram; EIC: extracted ion chromatogram; RT: retention time.

The enzymatic activities of the CsTPSs were tested with an in vitro assay, using GPP, FPP, GGPP, and neryl pyrophosphate (NPP) as substrates. CsTPS3GT was found to react only with FPP to form sesquiterpene (Z)-γ-bisabolene, while CsTPS4WS reacted exclusively with GPP to form linalool, a monoterpene alcohol, as reported for CsTPS29 (Figure 7a,b; [29]). As there are two natural enantiomers for linalool (R and S) [34,35], CsTPS4WS was further confirmed to be (R)-linalool synthase via chiral gas chromatography (Figure 7c). On the other hand, CsTPS5TH produced the sesquiterpene (E)-nerolidol with only FPP, thus confirming it as an (E)-nerolidol synthase (Figure 7d). CsTPS activity was further evaluated in vivo by transiently expressing them in the leaves of *N. benthamiana*. Consistent with the in vitro results, the characteristic peak of (R)-linalool with CsTPS4WS at 3 dpi (days post-infiltration) was detected. Similarly, CsTPS3GT and CsTPS5TH were confirmed to produce (Z)-γ-bisabolene and (E)-nerolidol, respectively (Figure 7e–g).

## 3. Discussion

This study provides clear evidence at the transcriptome level that illuminates the holistic and concerted adaptations of metabolism in Cannabis trichomes to facilitate cannabinoid and terpene production. For example, our findings on the importance of enhanced and coordinated AcCoA production in Cannabis trichomes is supported by studies in various organisms; an increase in AcCoA supply via overexpression of *PDH* and *AcCoA synthetase* (*ACS*) improved IPP precursor biosynthesis through the MVA pathway [24], while the enhancement of pyruvate level by up-regulating pyruvate kinase expression (e.g., *PK* and *PK2*) led to improved AcCoA availability, promoting terpene production [36]. The excessive demand of NADPH for secondary metabolite production was also supported by the increase in expression (D-value) of the gene encoding the ME (NADP+) enzyme (Figure 2a), which was the largest among the primary pathways. Consequently, the MDH reactions that compete for the same malate substrate to generate NADH instead were likely reduced. The phenomena of swapping NADH for NADPH production in Cannabis trichomes was repeated with the four-fold increase in the expression of the gene for the GAPN enzyme, which re-routed G3P away from the GAPDH and PGK reactions that produce NADH and ATP as an alternative. The findings further indicate that, if inappropriately dealt with, NADPH deficiency can be a major production bottleneck in the Cannabis plant. In this regard, the provision of NADPH and the other uncovered carbon substrates (G3P, pyruvate, and AcCoA) has been repeatedly demonstrated to result in high terpenoid productivity by engineered *Escherichia coli*, with a mass yield of up to 45% [36,37,38,39]. Thus, the current analysis newly demonstrates similar convergence in the adaptations of the Cannabis plant to enhance secondary metabolite yields. In addition, it was also found that the NAD(P)H-producing pathway, with enhanced *ME (NADP+)* expression (Figure 2c), possibly constitutes a modified acetate fermentation pathway. Firstly, PDH, PDC, and ALDH have functional roles in the pathway [40], and PDC is known to be induced in an anoxic environment [41]. When the fate of acetate (as the end metabolite of the pathway) was investigated, there was no indication of its being recycled to regenerate AcCoA [42] due to low *ACS* expression. Thus, the metabolite could be secreted in a fermentative manner. Furthermore, the glandular trichomes of the peppermint plant were also recently shown to exhibit fermentative metabolism [43], thus suggesting a similar feature in the Cannabis plant, for which the glandular trichomes of female flowers are the major sites of cannabinoid and terpene production [44]. This suggests that an anaerobic environment may not be required for the fermentation, as the trichome cells could just be prioritizing usage of their enzymatic capacity [45,46] for secondary metabolite production, over acetate recycling. Furthermore, the recycling is unnecessary, as carbon supplies are expected to be plentiful, given the productive role of the trichomes.

Furthermore, the current analysis highlights the heightened capacity of the MEP pathway compared to the MVA pathway in the trichomes, as suggested by the up-regulation of more genes encoding biosynthetic enzymes catalyzing rate-limiting reactions, and further characterized by larger fold changes and increased expressions (Figure 3a). This could be explained by the increased requirement for GPP as a precursor for monoterpene as well as cannabinoid biosynthesis in the fully matured flowers. Consistently, studies of other cultivars in a similar flowering stage also found genes encoding enzymes in the MEP pathway to be more highly expressed than those in the MVA pathway [47,48]. Genes in the latter pathway are instead more highly expressed during the early stage of flowering [48], in line with the larger sesquiterpene output reported during this time [49]. In addition, the exchange of isoprenoid precursors between two pathways is known to be possible [27], which further raises the possibility of the MEP pathway replenishing the MVA pathway for sesquiterpene biosynthesis, subject to the limited physical capacity of the chloroplast. Given the intense selection pressure for secondary metabolite production in the cultivars, such an adaptation would also benefit from the higher stoichiometric yield [50,51] and lower oxygen requirement [26] of the MEP pathway. The coordination of the pathways has also been previously proposed in a study, which established a negative correlation between *HMGR* expression in the MVA pathway and the MEP module comprising *CMK*, *MCS*, and *HDS* [52], which is consistent with the findings in this work. HMGR is further known to be a rate-limiting enzyme for the MVA pathway in many plants, whereas DXS and DXR are similarly so in the MEP pathway [53,54]. Although the bottlenecked production of limiting enzymes may be overcome by their over-expression, as demonstrated in many plants, the relevant research has not yet been carried out for Cannabis, to the best of our knowledge.

To illustrate the application of the current findings for crop selection to achieve high yields, a comparison of the expressions of pivotal enzyme genes, which were identified in commonly used cultivars, was pursued. There were large variations in their expressions and consequently in the implied production capacities of the cultivars. For example, there were 3.3-, 1.9-, 2.6-, and 1.8-fold differences in expression for *OAC*, *APT*, *THCAS*, and *CBDAS* among five cultivars (Figure 4a), thus exemplifying the need to profile cultivars molecularly. Although the WS and TH cultivars were easily identified as high and low producers, respectively, based on their highly consistent expression profiles, it is less clear how the other cultivars ought to be classified—a situation further exacerbated by the lack of chemical profiles for evaluation. Thus, further studies are needed to determine the relative utilities of key identified enzymes in discerning cultivar outputs.

Transcriptomics data are also highly useful for mining uncharacterized but highly expressed TPSs, which may contribute to the characteristic terpene repertoire of cultivars. Of the three genes picked out in this study, CsTPS5TH was experimentally verified to be an (E)-nerolidol synthase. On the other hand, CsTPS3GT and CsTPS4WS were initially described as mono-TPS1 (MTS1) and TPS9 in the cs10 Cannabis reference genome. However, in this work, CsTPS3GT and CsTPS4WS were found to synthesize a sesquiterpene (Z)-γ-bisabolene and a monoterpene (R)-linalool, respectively. Such misannotation of MTS1 underscores the challenge of using in silico assays for evaluating TPS function. Previously, an apple TPS was also mischaracterized as being most similar to mono-TPS due to the presence of the ‘RR(X8)W’ motif near the N-terminus [55]. However, it lacked the plastid transit peptide associated with mono-TPS, just like CsTPS3GT. Similarly, CsTPS4WS was initially considered to be capable of monoterpene cyclization because of the presence of the ‘RR(X8)W’ motif. However, it was found that it could only produce an acyclic monoterpene alcohol, (R)-linalool, using both in vitro and in vivo assays in this study. Given that the two natural enantiomers of linalool have distinct olfactory qualities, CsTPS4WS may contribute to the lavender-like smell of Cannabis with its production of (R)-linalool [56], rather than the floral and petitgrain-like scent of (S)-linalool [57]. This study also further affirms that minute amino acid differences can result in significantly different TPS activities. For example, although CsTPS5TH and CsTPS35 share 98.7% similarity at the amino acid level, CsTPS5TH could only produce nerolidol from FPP, which is unlike CsTPS35, which can synthesize both linalool from GPP and (E)-nerolidol from FPP. Similarly, CsTPS4FN and CsTPS9FN form different products [58], despite being 97% identical in their amino acid sequences.

## 4. Materials and Methods

### 4.1. Plant Material, Trichome Isolation, and RNA Isolation

All plants were grown indoors in a growth chamber using Coco coir–perlite (3:1) growing medium. The photoperiod was maintained under a 16 h light/8 h dark cycle during vegetative phase and a 12 h light/12 h dark cycle during generative phase to induce flowering. The ambient temperature was maintained at 28 °C. Flowers from CD, GT, WS, TH, and HB were collected at the 10th, 9th, 9th, 9th and 10th weeks, respectively. Trichomes from the fully matured female flowers of five Cannabis cultivars were separated by soaking the flowers in RNAlater solution with crushed ice, with continuous shaking for 5 min. The solutions were then passed through differently sized cell strainers to separate trichomes from other cell debris [58,59]. To obtain stem samples, trichomes were brushed off from the stems of HB cultivar plants; they were few in number and had very low levels of secondary metabolites, unlike the trichomes of flowers [60]. The collected trichomes and brushed stem samples were then sent to SeqMatic LLC (Fremont, CA) for RNA isolation and sequencing. Total RNAs from Cannabis trichomes were isolated using the Spectrum^TM^ Plant Total RNA Kit (Sigma-Aldrich), according to the manufacturer’s instructions, and their RIN scores were found to range between 5 and 8. The lower RIN scores of trichomes were due to their high metabolite contents. One biological replicate was used for transcriptomic analysis of the stem tissue and the trichomes of each cultivar. It is important to note that one biological replicate of the trichome consists of thousands of trichomes pooled together. *Nicotiana benthamiana* plants were grown in a greenhouse under long-day conditions (16 h under light/8 h in darkness) for 4 weeks before using them for subcellular localization experiments and in vivo TPS assays.

### 4.2. RNA Library, RNA-seq, Pre-Processing, and Quality Control

RNA libraries were constructed according to Illumina’s TruSeq strand-specific protocol, with a median insert size of ~230 bp (~100–350 bp). They were then sequenced using HiSeq 2500@PE100 platform (Illumina, San Diego, CA). Adaptor sequences were removed from the reads using Trimmomatic [61], while the quality of the reads was maintained by trimming using the ‘adaptive quality trimming’ algorithm [62]. The latter strikes a balance between quality score and sufficient read length, which is necessary for unambiguous mapping to the genome. Resulting reads < 36 bp were deemed to cause unspecific mapping and were thus discarded. Furthermore, only reads with a mean Q-score > 25 were retained, which translated to <1% error on average for each base pair. Upon completion, quality-control checks using FastQC did not reveal any issues with the final pre-processed reads.

### 4.3. Reference Genome Mapping and GC-Bias Correction

SALMON [63] was used to quantify transcript expression from the pre-processed reads in a rapid and memory-efficient manner by coupling quasi-mapping onto a reference transcriptome with a read count inference procedure. In doing so, it considers the specific experiment attributes and corrects their biases, including GC contents, to provide a more accurate estimate of the read counts. The latest cs10 transcriptomic and genomic sequences (https://www.ncbi.nlm.nih.gov/genome/?term=txid3482[orgn], accessed on 18 October 2020) were used as references for transcriptome mapping and identification of genomic decoys to prevent spurious mapping, respectively. A read count table based on transcripts was produced as a result.

### 4.4. Gene-Level Expression, Normalization, and Quality Control

Tximport [64] was used to sum up transcript read counts at the gene level. Following this, read counts were normalized using the TMM method and further divided by gene length to enable comparison across samples [65]. The TMM method normalizes the total RNA amount across samples and assumes that most genes are not differentially expressed. As such, it allows for the comparison of diverse samples, such as from different batches and tissue types, in contrast to within-sample normalization methods, such as RPM, FPKM, and TPM. Principal component analysis was then conducted on the gene expression dataset to assess whether there were any trichome sample outliers.

### 4.5. Differential Expression Analysis

The NOISeq algorithm [66] was used for the identification of DEGs, as it allows for a good control of false positives among lowly expressed genes by considering both M- and D-values. This is because lowly expressed genes tend to have high absolute M-values due to noise and may be construed wrongly as differentially expressed; however, by considering their low D-values, the false positives can be picked out statistically in a robust manner. This is especially applicable in discerning DEGs in the secondary pathways of plants (e.g., Cannabis plants) that may be lowly expressed but are biologically relevant. An 80% probability of being a true positive is used as the threshold for flagging a gene as differentially expressed. For genes having multiple genomic loci contributing to their expression, the percentage increase in trichome expression over the stem tissue for each gene can be determined by first taking the expression value of each locus in the trichomes as the average of all 5 cultivars. Then, the sum of all loci values can be taken to be the expression value of the gene. Gene expression in the stem tissue was similarly evaluated and then subtracted away from the corresponding expression in the trichome to determine the increase in expression. Gene expression increase was then reported as a percentage of its expression in the stem.

### 4.6. Hierarchical Clustering and Venn Diagram Analysis of DEG

Hierarchical clustering was carried out in R using complete linkage, and 1 − ρ^2^ as a measure of dissimilarity between all pairwise genes, where ρ is the Pearson’s correlation between the log_2_ fold-change (FC) values of pairwise genes. For representation of the clustering in heatmaps, the log_2_ FC values of each gene were centered and scaled based on the cultivars. The Venn diagram analysis was conducted online (http://www.interactivenn.net/) [67].

### 4.7. Gene Mapping to Metabolic Pathways

To analyze gene expressions in the metabolic pathways, they were first identified by a BLASTX search of cs10 gene sequences against the NCBI database to identify their Viridiplantae protein homologs. The KEGG IDs of the latter were then mapped to the pathways using the Blast2GO [68] module in OmicsBox 1.3.11 software (https://www.biobam.com/omicsbox [BioBam Bioinformatics, Valencia, Spain], accessed on 12 November 2020). In this way, cs10 genes with membership in metabolic pathways were identified (Table S5). More were manually picked out by inspecting their cs10 descriptions.

### 4.8. Quantitative Real-Time PCR (qRT-PCR)

The quantity and quality of RNA were measured using a Nanodrop spectrophotometer (ND-1000, Thermo Fisher Scientific, Waltham, MA, USA), and approximately 500 ng of RNA was reverse transcribed to cDNA using an iScript^TM^ cDNA Synthesis kit obtained from Bio-Rad, Singapore. Expression levels of enzyme genes along the trichomes of five cultivars and the stem of the HB cultivar were analyzed using qRT-PCR. Primers for qRT-PCR were designed by exploiting the cDNA sequences obtained from the RNA-seq data. The qRT-PCR reactions were performed in a 384-well PCR plate using KAPA SYBR fast master mix (Roche, Singapore) and an ABI PRISM 900HT real-time PCR system. For a total PCR reaction of 5 µL, 0.3 µL of cDNA was used and the cycling profile was set at 50 °C for 2 min, 95 °C for 10 min, 40 cycles of 95 °C for 15 s, and 60 °C for 60 s. After thermal cycles, the dissociation analysis (melting curve) was carried out to confirm the specific amplifications of the PCR reaction by adding a profile of 95 °C for 15 s, 60 °C for 15 s, and 95 °C for 15 s. In the current study, *CsGAPDH* was used as an internal reference, due to its similar expression across the five cultivars. A non-template reference was included for each gene to eliminate the possibility of random genomic DNA contamination and primer dimer formation. SDS 2.4 software (Applied Biosystems) was used to analyze the obtained results. The threshold cycle (Ct) value of a gene is the cycle number required for the SYBR Green fluorescence signal to reach the threshold level during the exponential phase for detecting the amount of accumulated nucleic acid [69]. Comparative delta Ct values of target genes to *CsGAPDH* were taken as relative expressions among different tissues. The amounts of target genes, normalized to the *CsGAPDH* gene, were calculated by 2^−(Ct[target gene]-Ct[GAPDH])^. Error bars represent means ± SDs. All primers used in this study were designed manually and are listed in Table S1.

### 4.9. Phylogenetic Tree and Clustal Analysis

A phylogenetic tree was constructed using MEGA7 software (Version 7.0, Pennsylvania State University, PA, USA) [70] by the neighbour-joining method with bootstrap values of 1000 replicates. The required sequences were obtained from the NCBI database, with their accession numbers listed in Table S2. The deduced amino acid sequences of CsTPSs were aligned with other functionally characterized CsTPSs using Clustal W, with the following parameters: gap open: 10; gap extension: 0.1; protein weight matrix: Gonnet; penalties: on; gap separation: 4; cut off: 30%.

### 4.10. Subcellular Localization of TPSs

Full-length ORFs of *CsTPS3GT*, *CsTPS4WS*, and *CsTPS5TH* were cloned into pENTR vectors using the pENTR™/D-TOPO^®^ Cloning Kit (Thermo Fisher Scientific, Singapore) and transformed into XL1-blue competent cells. Plasmids from the positive clones were isolated and cloned into the destination vector pBA-DC-YFP [71], which contains the YFP in frame at the C-terminal and cauliflower mosaic virus (CaMV) 35S promoter, to generate CsTPS3GT-YFP, CsTPS4WS-YFP, and CsTPS5TT-YFP, respectively. The constructs were transformed into the *Agrobacterium tumefaciens* EHA105 strain by a heat shock method [72]. The transformed EHA105 cells were cultured at 28 °C and resuspended in a solution containing 100 µM acetosyringone, 10 mM MES (pH 5.6), and 10 mM MgCl_2_. The above mixture was incubated at room temperature for 3 h and later infiltrated into *N. benthamiana* leaves using a 1 mL syringe. After 2 d, the infiltrated leaves were excised, and the fluorescence signals were observed under a confocal scanning laser microscope (LSM 5 Exciter, ZEISS, Jena, Germany). All constructs were verified by DNA sequencing.

### 4.11. In Vitro and In Vivo TPS Assays

For the in vitro TPS assay, the recombinant protein with 6His-tag was cloned into the destination vector pDEST17 and expressed using the *E. coli* BL21 pLysS strain. A cell pellet from 5 mL of isopropyl β-d-1-thiogalactopyranoside (IPTG)-induced culture was passed through a His spin trap (GE Healthcare) to obtain the cell lysate. The assay was carried out by mixing 250 µL of 2× reaction buffer (50 mM HEPES (pH 7.4), 200 mM KCl, 15 mM MgCl_2_, 10% glycerol, 10 mM DTT) with 50 µg of cell lysate, 5 µg of the substrate (GPP, FPP, NPP, and GGPP) and massed up to 500 µL with 25 mM HEPES (pH 7.4) in an inert glass bottle. Then, 250 µL of hexane was added on top slowly and the reaction bottle was sealed with parafilm. After an incubation at 30 °C for 2 h, the reaction mixture was vortexed for 1 min and centrifuged at 1200 rpm for 30 min. The hexane layer was then transferred to a fresh GC bottle and subjected to GC–MS analysis.

For the in vivo assay, A. tumefaciens cultures harbouring plasmids 35Spro:CsTPS, 35Spro:HMGR, and silencing suppressor 35Spro:p19 were pelleted and resuspended in MMA (10 mM MES, 10 mM MgCl_2_, and 100 µM acetosyringone) solution to OD_600_ = 1. The solutions were then mixed or infiltrated separately into *N. benthamiana* leaves using a 1 mL syringe, and 2–3 infiltrated leaves were excised after 3 d and incubated with 500 µL hexane for 1 hr. The homogenized samples were then centrifuged for 10 min at 13,000 revolutions per min (rpm). The hexane layer was transferred to glass vials and analyzed using GC–MS (7890A with 5975C inert mass selective detector, Agilent Technologies, Santa Clara, CA, USA). Then, 2 µL samples were injected, and separation was achieved with a temperature program of 50 °C for 1 min, increased at a rate of 8 °C/min to 300 °C and held for 5 min, on a 30 m HP-5 MS column or CP-Chirasil Dex CB column (25 m × 0.25 mm, 0.25 μm film thickness) (Agilent Technologies, Santa Clara, CA, USA). The compounds were identified by comparison with the mass spectra reference library NIST MS 2014. The data were processed by MSD ChemStation Data Analysis (Agilent Technologies). The enantiomeric identity of linalool was confirmed by comparison with the GC data of (S)-linalool and (R)-linalool standards.

## 5. Conclusions

In this study, transcriptome data of the glandular trichomes of five Cannabis cultivars were generated and compared with those of stem tissue without trichomes, thereby uncovering the coordinated regulation of primary and secondary metabolic pathways in the trichomes, which is consistent with their high levels of secondary metabolite production. However, further studies on the effects of these gene regulations are still needed at the protein, metabolite, and phenotypic levels (functional genomics) to support our findings. We examined the DEGs between trichomes and an organ without trichomes (namely, the stem after removal of all trichomes)—this because we were focused on the metabolic adaptations required for secondary metabolite production, rather than the differences among various organs, which may have been too convoluted for the achievement of our objective. Certainly, future studies could be directed at similarly comparing different organs, for example, leaves, stems, and flowers, to elucidate other nuanced metabolic adaptations in the latter.

Recently, there has been also a lot of research on selecting and developing Cannabis cultivars with specific traits. In this regard, the availability of transcriptomic profiles will enable researchers to make progress by elucidating the relevant genes to be targeted. With this in mind, the data for the five cultivars can serve as resources for such work. In addition, the function of three TPS genes were characterized, identifying them as (R)-linalool synthase, (Z)-γ-bisabolene synthase, and (E)-nerolidol synthase, thus adding to our communal knowledge of the diverse repertoire of Cannabis TPSs. Altogether, this work will contribute to the effective genetic characterization, selection, and development of Cannabis cultivars.

## Figures and Tables

**Table 1:** RNA-seq read statistics.

Tissue	Cultivar	Raw Reads (mil)	Reads after QC (mil)	Mapped Reads (mil)	% Raw Reads after QC	% Raw Reads after Further Mapping
Stem	HB	248	228	200	92%	88%
Trichome	CD	273	261	227	96%	87%
Trichome	GT	256	249	215	97%	86%
Trichome	HB	260	251	215	97%	86%
Trichome	TH	273	262	229	96%	87%
Trichome	WS	277	267	235	96%	88%

## Data Availability

RNA sequencing data can be retrieved from the NCBI Sequence Read Archive (PRJNA706039). Sequence data for CsTPS3GT, CsTPS4WS, and CsTPS5TH have been deposited in GenBank under the accession numbers MW713051, MW713052, and MW713053, respectively.

## Supplementary Materials

The following supporting information can be downloaded at: https://www.mdpi.com/article/10.3390/ijms23158310/s1.
