# Supplementary material for: Comparative Transcriptome Analysis Reveals Coordinated Transcriptional Regulation of Central and Secondary Metabolism in the Trichomes of Cannabis Cultivars
Source: Int J Mol Sci. 2022 Jul 27;23(15):8310. doi: 10.3390/ijms23158310 (PMC9368916; doi:10.3390/ijms23158310)
Supplement: Supplementary file 1 [file ijms-23-08310-s001.zip › ijms-1759849-supp/ijms-1759849-supplementary tables.zip!/Table S1.pdf]

Table S1. List of primers used in this study.

**Name**	**Sequence (5' to 3')**	**Purpose**
CsTPS3GT_f	CACCATGACGCAGTTAGGAGTAATCTCCAGTAGTAC	Cloning
CsTPS3GT_r1	TTAAATTGGAATTGGATCGAAGAACATGTTGACAACTTTGGTCTTAAGATATC	Cloning
CsTPS3GT_r2	AATTGGAATTGGATCGAAGAACATGTTGACAACTTTGGTCTTAAGATATC	Subcellular localization
CsTPS3GT_qPCR_f	CATGCCTTGGATCTTCCACT	Quantitative PCR
CsTPS3GT_qPCR_r	AGAACCACTCCACCACCTTG	Quantitative PCR
CsTPS4WS_f	CACCATGTCATCGATTATTTATTCCCCGTTTACTTCTCTACTTC	Cloning
CsTPS4WS_r1	TTATTTTTTTATTTTAGTTTTTATGGTTGTATGTGAGTCTCCAAGTCCATTAATTGGTTTGG	Cloning
CsTPS4WS_r2	TTTTTTTATTTTAGTTTTTATGGTTGTATGTGAGTCTCCAAGTCCATTAATTGGTTTGG	Subcellular localization
CsTPS4WS_qPCR_f	GGAGGATGCCAAGATCTGAA	Quantitative PCR
CsTPS4WS_qPCR_r	AAATTTTCCCACCACCTTCC	Quantitative PCR
CsTPS5TH_f	CACCATGGCTTCTCCCTTTCTTTATGTCCCTTCTAAAC	Cloning
CsTPS5TH_r1	TTATATTGTTTGATGAGAGAGAAGTGAGTTGATATAATTCTGGAGGCC	Cloning
CsTPS5TH_r2	TATTGTTTGATGAGAGAGAAGTGAGTTGATATAATTCTGGAGGCC	Subcellular localization
CsTPS5TH_qPCR_f	AACATTCCAGGCCTTGTGTC	Quantitative PCR
CsTPS5TH_qPCR_r	GGCGTTCCCACTCATTTTTA	Quantitative PCR
CsGAPDH_qPCR_f	GCACCTATGTTCGTGGTTGG	Quantitative PCR
CsGAPDH_qPCR_r	ACGGTCTTCTGTGTTGCTGT	Quantitative PCR
