# Supplementary material for: Comparative Transcriptome Analysis Reveals Coordinated Transcriptional Regulation of Central and Secondary Metabolism in the Trichomes of Cannabis Cultivars
Source: Int J Mol Sci. 2022 Jul 27;23(15):8310. doi: 10.3390/ijms23158310 (PMC9368916; doi:10.3390/ijms23158310)
Supplement: Supplementary file 1 [file ijms-23-08310-s001.zip › ijms-1759849-supp/ijms-1759849-supplementary tables.zip!/Table S2.pdf]

Table S2. Accession number of terpene synthases used in Figure 5.

Gene	Genbank ID	Strain
CsTPS1FN	ARE72253.1	Finola
CsTPS2FN	ARE72261.1	Finola
CsTPS3FN	ARE72257.1	Finola
CsTPS4FN	ARE72260.1	Finola
CsTPS5FN	ARE72256.1	Finola
CsTPS6FN	ARE72259.1	Finola
CsTPS7FN	ARE72250.1	Finola
CsTPS8FN	ARE72252.1	Finola
CsTPS9FN	ARE72251.1	Finola
CsTPS14CT	QCY41294.1	Canna Tsu
CsTPS15CT	QCY41293.1	Canna Tsu
CsTPS16CC	QCQ18307.1	Cherry Chem
CsTPS18VF	QCY41292.1	Valley Fire
CsTPS19BL	QCY41291.1	Black Lime
CsTPS20CT	QCY41290.1	Canna Tsu
CsTPS3GT	MW713051	Ghost OG x NBK
CsTPS4WS	MW713052	Westside OG
CsTPS5TH	MW713053	Tahoe OG x NBK
CsTPS31	QLC36839.1	Purple Kush
CsTPS29	QLC36833.1	Purple Kush
CsTPS35	QLC36840.1	Lemon Skunk
CsTPS32	QLC36849.1	Purple Kush
CsTPS34	QLC36841.1	Purple Kush
CsTPS36	QLC36836.1	Purple Kush
CsTPS65	QLC36850.1	Purple Kush
CsTPS66	QLC36851.1	Purple Kush
CsTPS5(PK)	QLC36846.1	Purple Kush
CsTPS16	QLC36843.1	Purple Kush
CsTPS17	QLC36835.1	Afghan Kush
CsTPS18	QLC36838.1	Chocolope
CsTPS20	QLC36834.1	Lemon Skunk
CsTPS21	QLC36848.1	Afghan Kush
CsTPS22	QLC36842.1	Purple Kush
CsTPS23	QLC36845.1	Chocolope
CsTPS25	QLC36837.1	Lemon Skunk
CsTPS26	QLC36844.1	Purple Kush
CsTPS28	QLC36847.2	Purple Kush
