# Supplementary material for: Comparative Transcriptome Analysis Reveals Coordinated Transcriptional Regulation of Central and Secondary Metabolism in the Trichomes of Cannabis Cultivars
Source: Int J Mol Sci. 2022 Jul 27;23(15):8310. doi: 10.3390/ijms23158310 (PMC9368916; doi:10.3390/ijms23158310)
Supplement: Supplementary file 1 [file ijms-23-08310-s001.zip › ijms-1759849-supp/ijms-1759849-supplementary tables.zip!/Table S7.pdf]

Table S7. Percentage by mass for selected secondary metabolites in the five Cannabis cultivars.

Metabolite	Cultivar				
	**WS**	**CD**	**HB**	**TH**	**GT**
THC	21.9	19.0	14-22	14-21	15-20
CBD	0.06	<1	<1	<1	<1
CBDA	1.32	0.1	-	-	-
β-Caryophyllene	0.89	0.78	0.13	0.17	0.15
Limonene	0.54	0.10	0.18	0.39	0.27
β-Myrcene	0.28	0.09	0.50	0.57	0.62

Numbers represent percentage of metabolites. "-" refers to no information available. The data was obtained from two websites: https://www.hytiva.com/ and https://mcrlabs.com/.
