# Supplementary material for: Comparative Transcriptome Analysis Reveals Coordinated Transcriptional Regulation of Central and Secondary Metabolism in the Trichomes of Cannabis Cultivars
Source: Int J Mol Sci. 2022 Jul 27;23(15):8310. doi: 10.3390/ijms23158310 (PMC9368916; doi:10.3390/ijms23158310)
Supplement: Supplementary file 1 [file ijms-23-08310-s001.zip › ijms-1759849-supp/ijms-23-08310-s001.zip!/ijms-1759849-supplementary.pdf]

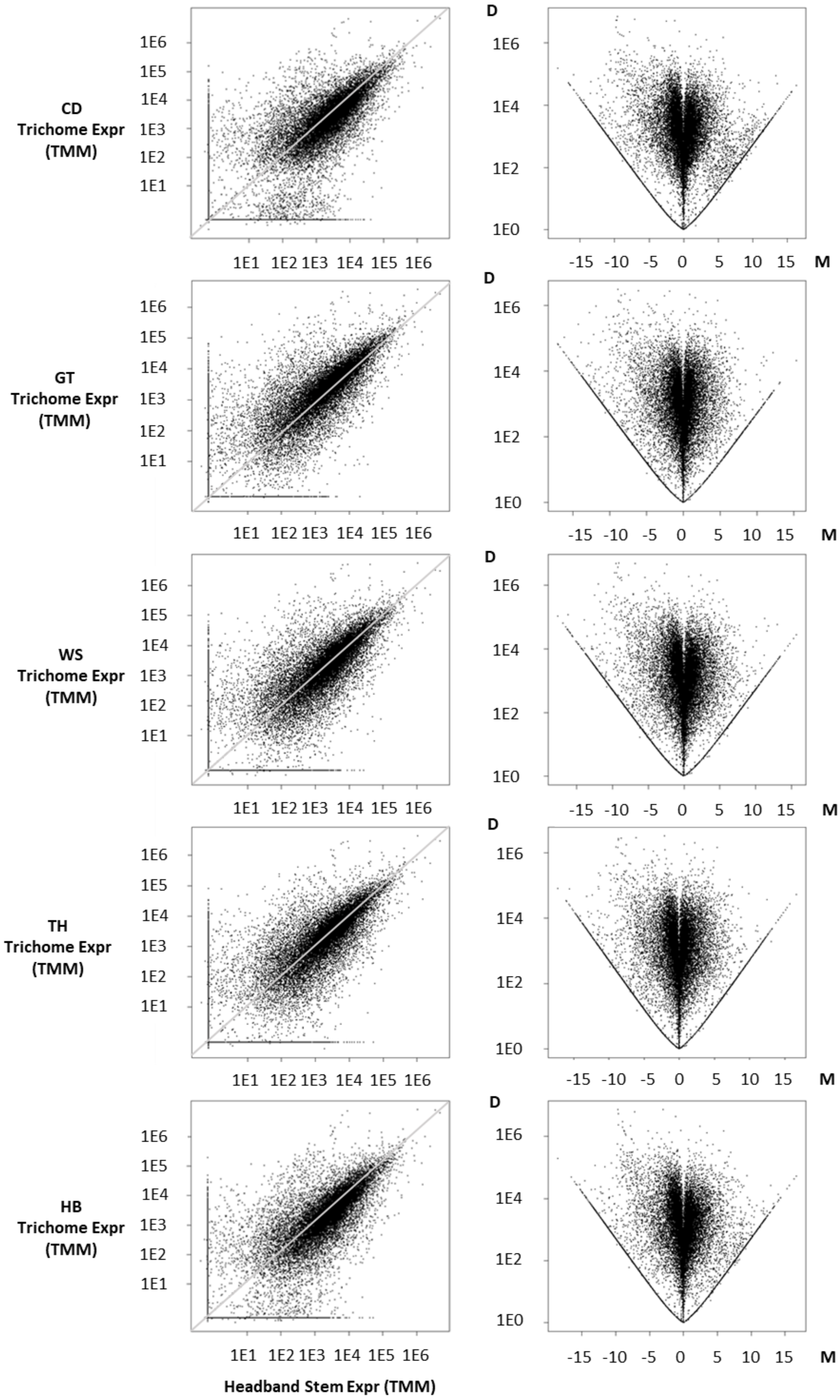


**Figure S1.** Scatter plots of global gene expression values for the trichomes of each cultivar versus those of stem tissue (left) and their corresponding D-values – M-values plots (right).

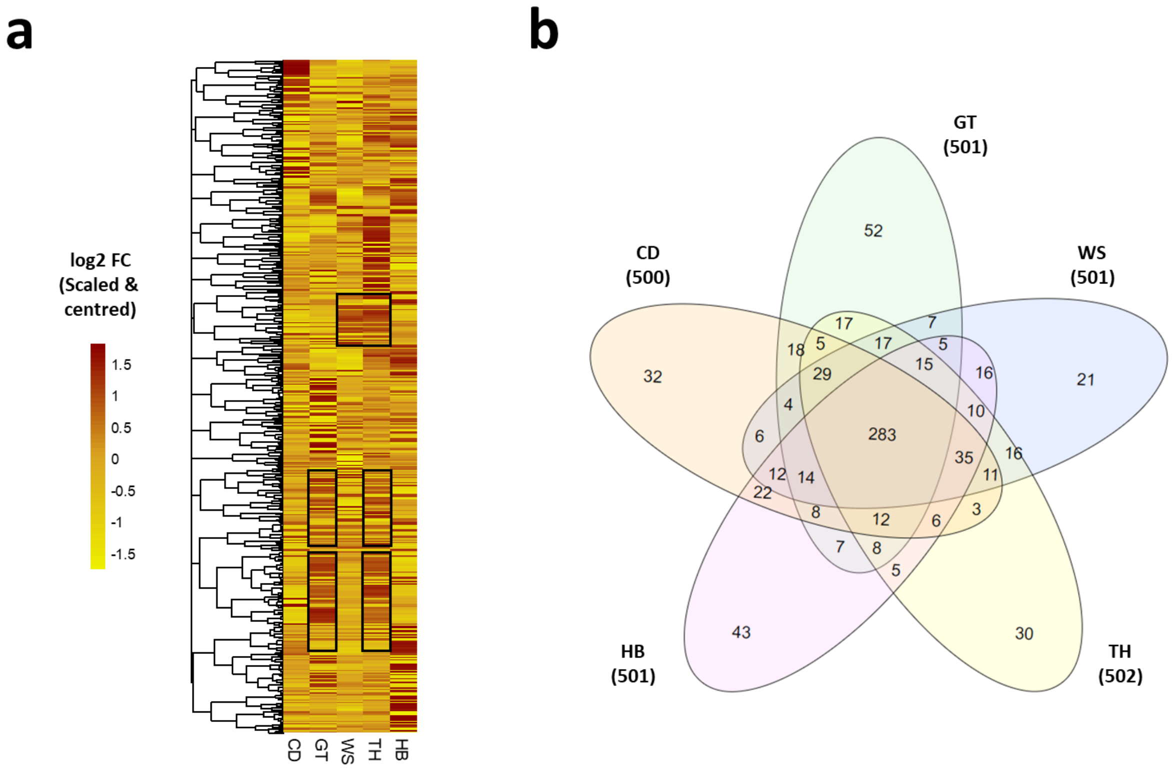


**Figure S2.** Exploratory analysis of DEG similarity among cultivars. a. Hierarchical clustering of $log_2$ fold-change value for the top 500 DEG of each cultivar (trichomes compared to stem tissue). Cells with black border denote similar expression pattern among corresponding cultivars. b. Venn diagram showing set analysis of the same DEG. Their number may exceed 500 for each cultivar due to ties in probability value.

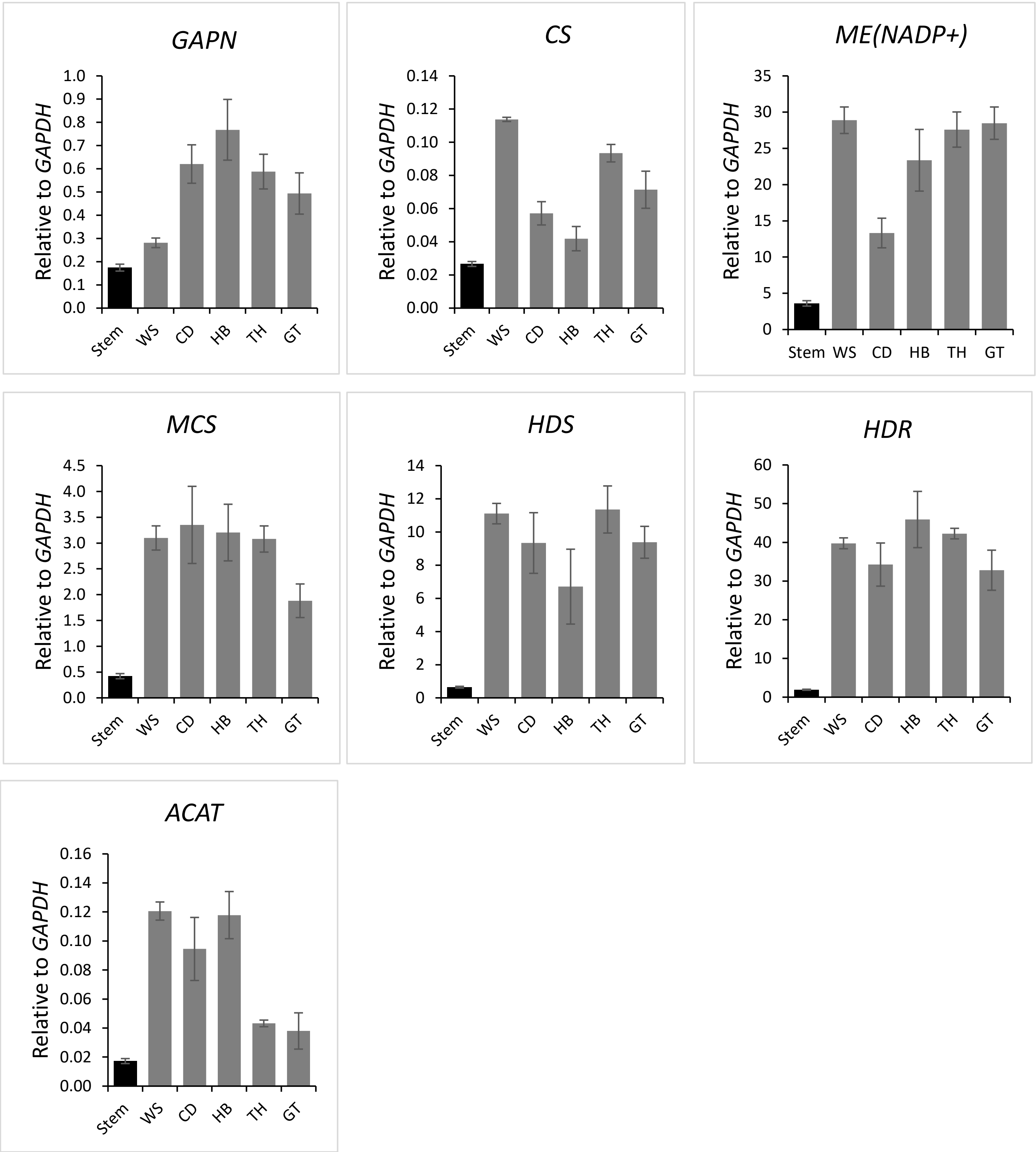


**Figure S3.** Expression of *GAPN*, *CS*, *ME(NADP+)*, *MCS*, *HDS*, *HDR* and *ACAT* in the trichomes of five cultivars and stem of HB cultivar.

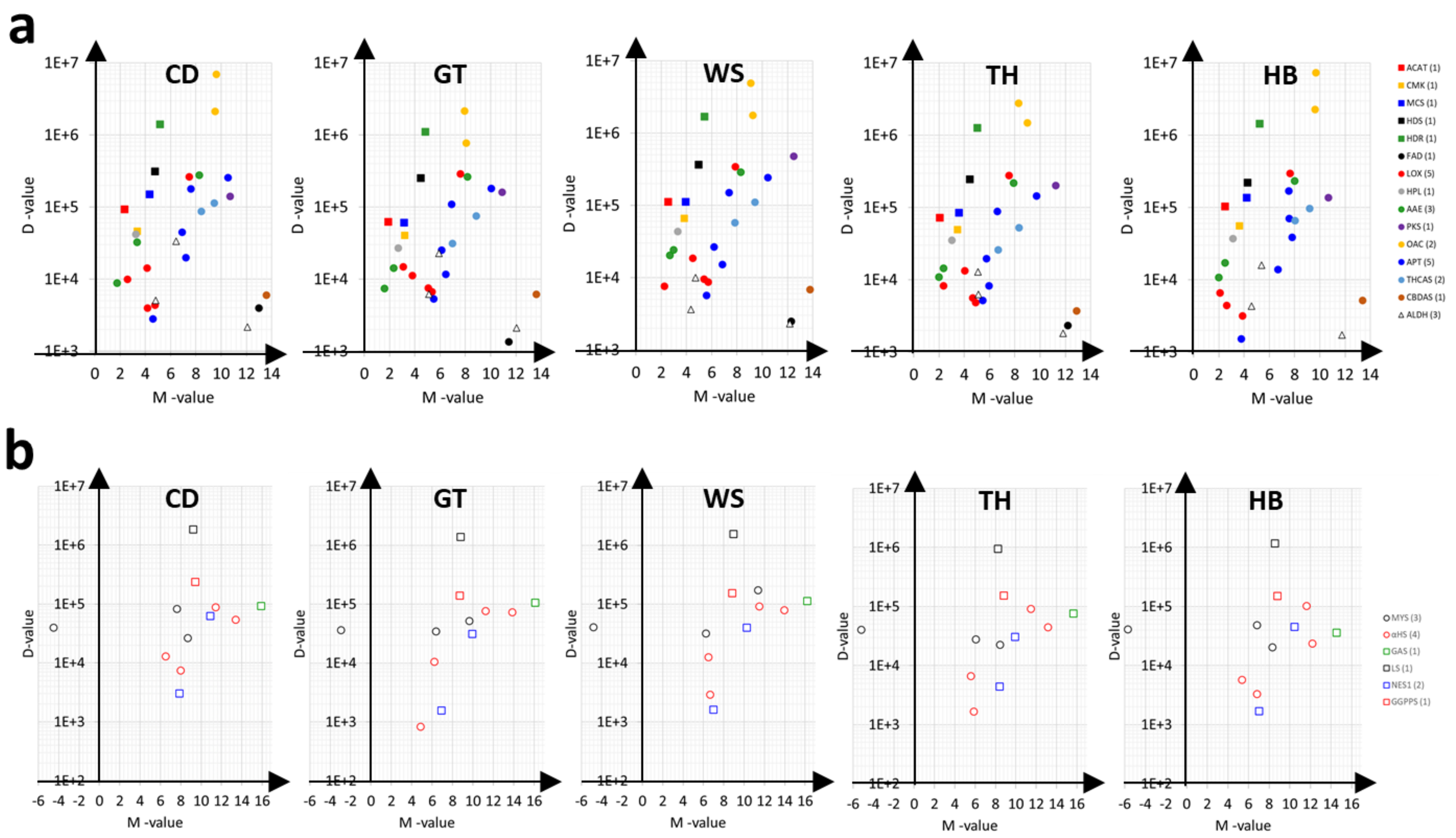


**Figure S4.** Corresponding plots of Fig. 3 for individual cultivars in the primary (a) and secondary (b) metabolic pathways.

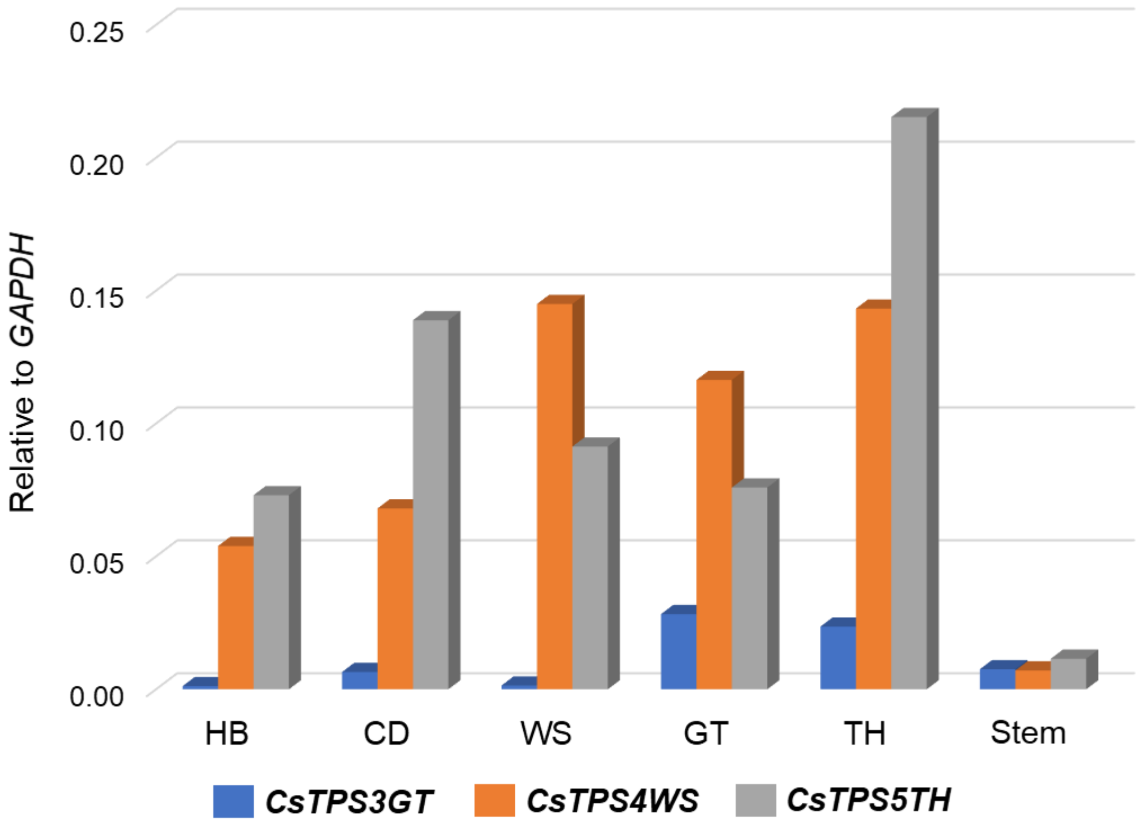


**Figure S5.** Expression of *CsTPS3GT*, *CsTPS4WS*, and *CsTPS5TH* in the trichomes of five cultivars and stem of HB cultivar.

```
                                                      RR(X8)W
CsTPS3GT  -----------MTQLGVISSSTP----------IFKDQPAAIVR----RSGNYKPTLWDAHFFQSLQVIY  45
CsTPS4WS  MSSIIYSPFTSLLPLKPISSASSTATINTRLKSRFRSSILVVLRPQQRRSAKYHPTVWENKHIDSFFTPY  70
CsTPS5TH  ----------------------------------MASPFLYVPS----KHGNGLQSFTDPFYMEYTRNIR  32

CsTPS3GT  TEESYGKRINELKEDVRRILEKEAENPLVKLEQINDLSRLGISYHFEDQIKTILN--------LTFNNNN  107
CsTPS4WS  NYELHSERLQELKQITSTSLR-TTKDPCILLKLIDSIQRLGLEYHFENEIEDAVS--------FIYAHND  131
CsTPS5TH  EMKNVMNSIILADDDYR-------ERSIEALNLVNAVLRVGIDYHVQDEIKSILEREHIIFSDHISNQYF  95

CsTPS3GT  ALWKKDNLYATALHFKLLRQYGFSPVSSEVFNAFKNEKKEFKESLSKDVKGMVCLYEASFYSFRGEPILD  177
CsTPS4WS  QTTSN-DLFMTALRFRILRQHGLF-VGSDVFDRFRGRDGKFLDSLSSNKHGILSLYEASHLGMPEENVLE  199
CsTPS5TH  NNINQDHLYEVSLRFRLLRQGGYDVSPDVFNELMKDKKGNFNVLVEEDREGLRELFEASQVRIEGEEVLE  165

CsTPS3GT  EARDFTTKHLKQYLMTRQSQFTRVDHHDDDDHDLVKLVEHALDLPLHWRLPRLEARWFIDMYAERNYDMN  247
CsTPS4WS  EAKSFTTKRLRYFSAG-----------KMDTTLFGKQVKQSLEVPLYWRMPRSEARNFIDLYQMDETKS-  257
CsTPS5TH  EAEVFSGGHLKEWANLHR------------HTSEARSIQLTLDQPCHKSLARVTSPNFLDSFSANTTTID  223

CsTPS3GT  ------PTFLDFAKLDYNFVQSAYQKELKYISRWWSGSRLTERLPFARDRVVEIFYSAVALKYEAEFGFV  311
CsTPS4WS  ------VTLLELAKLDYNLVQSVHQNELKELGRWWDDLGFKKNLPFARDRVVENYLWAMGIVSEPQFSKC  321
CsTPS5TH  QGWTWMTLLNKLVTMDFKIVQSIHQREIVLVSKWWKELGLAEELKFARDQPPKWYLWTVASLPDPSLSEE  293
                         DDXXD
CsTPS3GT  RTVMTKIGLLLTLMDDIYDVYGTLDELQLFLEAIERWNINEL-DQLPDYMKILFVAFYNNVNEISYYVLK  380
CsTPS4WS  RIGLTKFVCILTAIDDVYDIYGSLDELELFTNAVESWDIRAIRDEFPLYLKTCYLGMLNFGNEVIDDVLQ  391
CsTPS5TH  RIELTKPISLVYIIDDIFDVYGTLDELTLFTDAVNNWEIKEQ---LPDYLKICFKALDDITNKISYVVYR  360

CsTPS3GT  ENGIHTIKYLKKALGDLCKCYMEEAKWFHSGHIPSLEEFIENGWKSITIPLCLIYHYCLITT---SITEQ  447
CsTPS4WS  NHGLNISSYIKEEWLNLCKSYLVEARWFYNDYTPSLNEYLENSSTSVGGHAAIVHACILLLDGSIPETLL  461
CsTPS5TH  KHGWNPIDSLRKSWGKLCNAFLLEAEWFGCGKLPNEEEYLKNAIVSSGVHVVLVHMFFLLGEG---ISME  427
                                 NSE/DTE
CsTPS3GT  DMEHLLQYPTILRVSGTVFRFIDDLGTSSDELERGDNPSSIQCYMREKGVSENE-SREHIWNLISEGWKE  516
CsTPS4WS  DYNFNHFHSKLIYWSSLITRLSDDLGTSKDELKRGDVKKSVECYMAEKGIWEEEEAINHIKELRRNSWKM  531
CsTPS5TH  AVNLLDNIPGLVSSTAAILRLWDDLGSAKDENQNGHDGSYVECYMKRHKECSMGEAREQVIRMIKNEWER  497

CsTPS3GT  IN-EVKASTSPYSQVFIESAIDFVRGAMEMYHKGDGFGTNQDRYLKTKVVNMFFDPIPI-----------  574
CsTPS4WS  VNKEIIIGNNCLPKIMVKMCLNMARTAQFIFQHGDGIGTSTG-ATKHRLASLIVKPVPIIDPCSKPINGL  600
CsTPS5TH  LNKECFS-SKHFPMCFRKGCLNAARMVPVMYDYDD---HHRLPGLQNYINSLLSHQTI------------  551

CsTPS3GT  -------------                                                           574
CsTPS4WS  GDSHTTIKTKIKK                                                           613
CsTPS5TH  -------------                                                           551
```

**Figure S6.** Amino acid sequence alignment of CsTPS3GT, CsTPS4WS, and CsTPS5TH. Conserved regions are highlighted in black, while similar regions are in gray. Amino acids that are usually conserved in CsTPSs, are shown in red boxes. 'WW': conserved tandem tryptophan motif; 'RR(X8)W': tandem arginine motif of mono-TPS; 'DDXXD': divalent metal ion-assisted binding and ionization motif. The plastid-targeting transit peptide is underlined.

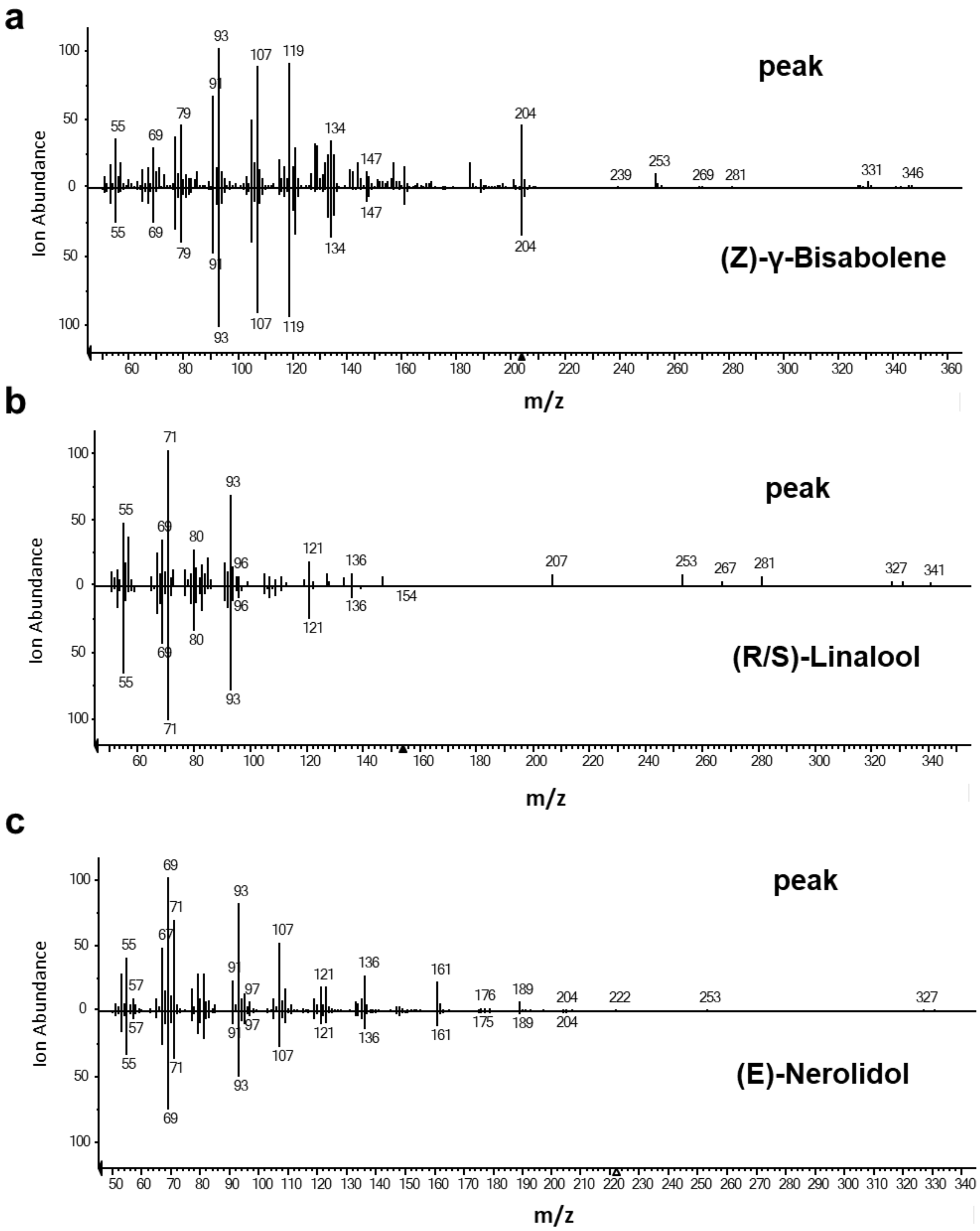


*m/z*: mass-to-charge ratio

**Figure S7.** Mass spectra of the products produced by CsTPS3GT (a), CsTPS4WS (b), and CsTPS5TH (c).
